# A cluster-based SMOTE both-sampling (CSBBoost) ensemble algorithm for classifying imbalanced data

**DOI:** 10.1038/s41598-024-55598-1

**Published:** 2024-03-02

**Authors:** Amir Reza Salehi, Majid Khedmati

**Affiliations:** Department of Industrial Engineering, Sharif University of Technology, 9414 Azadi Ave, P.O. Box 11155, Tehran, 1458889694 Iran

**Keywords:** Computational science, Computer science, Scientific data, Cardiovascular biology

## Abstract

In this paper, a Cluster-based Synthetic minority oversampling technique (SMOTE) Both-sampling (CSBBoost) ensemble algorithm is proposed for classifying imbalanced data. In this algorithm, a combination of over-sampling, under-sampling, and different ensemble algorithms, including Extreme Gradient Boosting (XGBoost), random forest, and bagging, is employed in order to achieve a balanced dataset and address the issues including redundancy of data after over-sampling, information loss in under-sampling, and random sample selection for sampling and sample generation. The performance of the proposed algorithm is evaluated and compared to different state-of-the-art competing algorithms based on 20 benchmark imbalanced datasets in terms of the harmonic mean of precision and recall (F1) and area under the receiver operating characteristics curve (AUC) measures. Based on the results, the proposed CSBBoost algorithm performs significantly better than the competing algorithms. In addition, a real-world dataset is used to demonstrate the applicability of the proposed algorithm.

## Introduction

The necessity to comprehend massive, complex, and information-rich datasets has now grown in various domains due to the data’s ever-expanding scope. In today’s competitive environment, the capacity to obtain valuable knowledge from these vast amounts of data is becoming increasingly crucial, and data mining provides this opportunity. The basis of data mining is provided by various fields, including machine learning, artificial intelligence (AI), probability, and statistics ^1^. Classification and clustering are two essential techniques used to extract knowledge from data. The capacity of these algorithms to identify hidden patterns and build models from data gives them their strength and efficiency. Clustering is an algorithm for extracting knowledge by grouping the data based on informative patterns to make observations within the same group as similar as possible and observations between separate groups as dissimilar as possible. Instead, the classification techniques attempt to predict categorical classes, such as heart disease conditions, based on a trained model^2^. If fact, the purpose of classification is an accurate prediction of the class label of observations. However, the class imbalance issue is one of the main challenges of data mining techniques that strongly affects the performance of the classifiers and hence, has received significant attention from researchers. This issue happens when the class label of observations is not equally distributed, and accordingly, there exists a majority class and a minority class. This imbalance may be attributed to diverse real-world factors, including infrequent incidents, limitations in resources, or biases in data collection^3^. In this regard, most of the observations belong to the majority class, while a few belong to the minority class^4^. The results obtained from the imbalanced data will not be highly reliable^5^, and most of the algorithms tend to bias toward the majority class and ignore the importance of the minority class. However, it should be noted that accurate prediction of the minority class is even much more important than the majority class. In other words, the minority class is, in most cases, the most important class that the experts are interested in. Numerous machine learning applications grapple with the significantly imbalanced datasets, such as detecting fraud in transactions, identifying faults, and making medical diagnoses. In these scenarios, the tolerance for predicting false positives is frequently higher, as the focus is typically on the minority class, making it more acceptable to prioritize sensitivity over specificity^6^. Accordingly, it is necessary to apply data-balancing algorithms in these cases. In this regard, data-level techniques, algorithm-level algorithms, and hybrid algorithms are three types of algorithms developed for dealing with class imbalance issues^7^.

The data-level algorithms work based on modifying the distribution of data by sampling algorithms where over-sampling and under-sampling are the two main sampling algorithms. The purpose of the under-sampling technique is to reduce the observations of the majority class by randomly eliminating observations. However, the main drawback of this algorithm is the loss of information caused by removing a portion of the data, where some algorithms are proposed in the literature to deal with this problem^8^. Instead, in the over-sampling algorithm, some observations are duplicated to obtain a balanced data distribution. However, this procedure results in the overfitting problem on the training data, where numerous algorithms have been proposed in the literature to address this issue^9^. The algorithm-level algorithms are mainly based on upgrading the existing learners to reduce their distortions toward majority groups. This procedure necessitates a thorough understanding of the revised learning algorithm and a detailed identification of the reasons for its failure when mining skewed distributions^10^. The most well-known algorithm-level technique is cost-sensitive learning that rebalances the classes based on their costs, and it may be implemented in different ways, including re-weighting or re-sampling training samples in proportion to their costs, pushing the classifier decision boundaries away from high-cost classes in proportion to costs, and so forth^11^. Finally, the hybrid/ensemble algorithms are based on implementing both data-level and algorithm-level algorithms to maximize their strengths and minimize their limitations. It should be noted that various data-level, algorithm-level, and hybrid algorithms have been proposed in the literature^12^. However, considering the superiority of hybrid/ensemble algorithms, a novel hybrid algorithm, entitled CSBBoost, is proposed in this paper to overcome the imbalanced datasets issues and improve the performance of the algorithm in classifying imbalanced data.

The remainder of the paper is organized as follows. In the next section, the algorithms presented in the literature for the classification of imbalanced data are presented. A brief explanation of some ensemble algorithms, including XGBoost, bagging, and the random forest, is provided in Sect. “Preliminaries”. The details of the proposed algorithm and its performance are presented in Sect. “The proposed algorithm”. The performance of the proposed algorithm is evaluated and compared to the competing algorithms in Sect. “Performance evaluation”. The proposed algorithm is applied to a real-world case study in Sect. “A case study”. Finally, the paper is concluded in Sect. “Conclusions”.

## Literature review

In this section, a thorough literature review of different algorithms proposed for dealing with imbalanced datasets is provided. Generally, there exist three categories of algorithms, including data-level techniques, algorithm-level algorithms, and hybrid algorithms, in the literature. These algorithms are discussed in detail as follows.

### Data-level methods

The data-level algorithms modify the distribution of data by sampling algorithms, including over-sampling and under-sampling. It should be noted that several research efforts have been devoted to under-sampling and over-sampling algorithms in the literature. In this regard, Tsai et al^13^. provided a novel under-sampling strategy that combines clustering analysis with observation selection. The clustering analysis is used to cluster identical observations in the majority class, and the observation selection algorithm is used to exclude unrepresentative observations from each category. Kubat and Matwin^8^ introduced the One Side Selection (OSS) algorithm focused on the effects of simple selection procedures that were modified to remove negative cases while maintaining all positives. The OSS under-sampling algorithm eliminates negative cases that are thought to be borderline, noisy, or redundant. The disadvantage of OSS is that a large number of negative cases are removed, which may degrade the classifiers’ performance. Guzmán-Ponce et al^9^. presented a cluster-based algorithm that uses DBSCAN to illustrate a filtering phase to detect and eliminate noisy negative observations, followed by a graph-based step that generates a representative sub-sample of the majority class with a pre-determined maximum imbalance ratio. To address the problem of losing data, it is necessary to specify which and how many observations should be sampled. To deal with this problem, Xie et al^14^. suggested a novel under-sampling algorithm called Progressive Under-sampling Algorithm with Density (PUMD) which instantly discovers observations of majority classes that are major components of data distributions and effectively determines the appropriate under-sampling size, efficiently downsizing datasets and removing observations of majority classes that are unimportant to classification tasks. On the other hand, Chawla et al^15^. proposed the synthetic minority oversampling technique (SMOTE) algorithm that employs interpolation between positive examples that are close together to produce new observations in the feature space. Several developments have been made in the SMOTE algorithm, including Borderline-SMOTE^16^, ADASYN^17^, FF-SMOTE^18^, and Cure-SMOTE^19^. Nekooeimehr and Lai-Yuen^20^ proposed a novel algorithm that employs a semi-unsupervised hierarchical clustering algorithm to cluster minority samples and adaptively calculates the size to over-sample each sub-cluster according to the classification complexities. In addition, Menardi and Torelli^21^ developed an over-sampling algorithm called Random Over Sampling Examples (ROSE), which is based on a smoothed bootstrap type of data re-sampling and is supported by well-known characteristics of kernel algorithms. Fonseca and Bacao^22^ proposed G-SMOTENC, combining G-SMOTE and SMOTENC to address imbalanced learning for datasets with both nominal and continuous features. The method demonstrated significant performance improvement compared to baseline methods across various datasets with varying characteristics.

### Algorithm-level methods

#### Cost-sensitive algorithms

The algorithm-level algorithms try to improve the performance of algorithms in classifying imbalanced datasets through modification of the learners. The cost-sensitive learning is the most well-known algorithm-level technique. At the same time, the one-class classifier ensemble is another algorithm-level algorithm that, in many circumstances, performs similarly to binary committees and can greatly surpass traditional algorithms in some more complicated scenarios^23^. Mienye and Sun^24^ presented robust cost-sensitive classifiers that alter the objective functions of some well-known algorithms, including logistic regression, decision trees, extreme gradient boosting, and random forest, to detect medical diagnoses reliably. Zhang et al^25^. proposed a new algorithm entitled cost-sensitive residual convolutional neural network (CS-ResNet) as an improved version of ResNet, where they added a cost-sensitive adjustment layer into the standard ResNet. In particular, CS-ResNet is optimized by minimizing the weighted cross-entropy loss function after assigning bigger weights to minority actual faults depending on the class-imbalance degree.

#### Ensemble algorithms

Ensemble algorithms such as bagging, Random Forest, and boosting integrate multiple classifiers to generate the result of the ensemble classifier in order to enhance the classification performance. To name a few research efforts in this field, Yin et al^26^. used the ensemble learning stacking algorithm to integrate four conventional algorithms, including k-nearest neighbors (KNN), support vector machine (SVM), deep neural network (DNN), and recurrent neural network (RNN). Arya and Hanumat^27^ suggested deep ensemble techniques for combining several base learners. Deep learning is used to improve the performance by obtaining lower-level information and passing them forward to the next layer in order to find higher-level attributes.

### Hybrid methods

The hybrid methods apply the algorithms of both data-level and algorithm-level methods in order to maximize their strengths and minimize their limitations. In this regard, Chawla et al^28^. proposed the SMOTEBoost algorithm as a combination of SMOTE and boosting algorithms. Freund and Schapire^29^ developed a boosting algorithm called AdaBoost using the multiplicative weight-update technique. The basic idea underlying boosting techniques is that one initially builds a model based on the training dataset and then builds a second model to correct the mistakes in the first one. This technique is repeated to reduce errors and generate the most accurate projected dataset. Seiffert et al^30^. introduced RUSBoost, a hybrid boosting algorithm for learning from biased training data. This algorithm is a more sensible and quicker alternative to SMOTEBoost. Díez-Pastor et al^31^. presented RBBoost, a new way to create ensembles of classifiers for two unbalanced class datasets. Each component of the RBBoost ensemble is trained using data from the training set and enhanced with SMOTE-generated synthetic observations. Rayhan et al^32^. developed an algorithm entitled Cluster-based Under-sampling with Boosting (CUSBoost) to address the issue of class imbalance. CUSBoost primarily clusters the observations of the majority class before performing random under-sampling, allowing the boosting algorithm (AdaBoost) to select instances from all regions of the dataset. Gong and Kim^33^ presented RHSBoost, which employs a hybrid sampling technique based on under-sampling and ROSE sampling. The AdaBoost algorithm is used as an ensemble technique in the proposed strategy. Rayhan et al^34^. introduced MEBoost, a novel boosting technique for unbalanced datasets. MEBoost combines two separate weak learners with boosting to enhance performance on unbalanced datasets. Zhao et al^35^. presented a weighted hybrid ensemble algorithm (WHMBoost) for classifying unbalanced data in binary classification cases. The proposed algorithm, within the context of the boosting algorithm, integrates two data sampling algorithms and two base classifiers. El Moutaouakil et al^36^. proposed Optimal Entropy Genetic Fuzzy-C-Means SMOTE (OEGFCM-SMOTE), for handling imbalanced datasets in classification problems. This method minimizes noise through an optimized combination of fuzzy clustering, SMOTE, and genetic algorithms, outperforming other oversampling techniques across various datasets and classifiers. Jia et al^37^. proposed a novel approach, TDMO, which leverages XGBoost and dynamic multi-dimensional oversampling to address imbalanced data issues. TDMO effectively filters out noise, evaluates class densities, and enhances the minority class, outperforming existing oversampling methods in classification results. Kumari et al^38^. proposed SMOTE-Stacked hybrid model (SmS) for early Polycystic Ovary syndrome (PCOS) diagnosis, combining SMOTE and stacking ensemble techniques. This model, utilizing classifiers like LR, SVM, DT, RF, NB, and AdaB, demonstrated promising results, where Stack-AdaB exhibited the most noteworthy performance on an imbalanced PCOS dataset. Guan et al^39^. proposed a novel solution to address imbalanced data classification challenges by introducing Extended Natural Neighbor (ENaN) without parameters, derived from Natural Neighbor (NaN). ENaN enhances the quality of synthetic examples in resampling methods like SMOTE, outperforming traditional approaches in improving sample distribution according to extensive experiments on synthetic and real-world datasets.

Despite many algorithms proposed in the literature to deal with imbalanced data, there are some disadvantages to these algorithms. In this regard,

- Over-sampling techniques increase the size of the dataset and make it challenging to execute learning operations on the dataset,
- Under-sampling techniques result in the loss of a significant amount of information,
- Duplication of samples during over-sampling results in overfitting on the training dataset,
- Considering the randomness of the under-sampling and over-sampling procedures, the chosen data may not accurately reflect the features of the dataset,
- Algorithm-level and ensemble algorithms individually do not result in a steady state performance and are sensitive to the imbalances in datasets.

A large amount of data in a dataset may be grouped according to how closely they resemble each other, and better outcomes may arise from balancing each group. Since generating new data in over-sampling and sampling from the majority class in under-sampling inevitably results in some flaws in the final model, ensemble techniques will be used in this paper for training classifiers based on balanced data. It should be noted that the suggested technique differs significantly from the current algorithms, where it offers a novel strategy for over-sampling and under-sampling to ensure that the best samples are included in the model. This research study aims to achieve the following objectives and contributions:

- A novel framework is proposed to address binary imbalanced learning. This framework involves the simultaneous utilization of undersampling and oversampling techniques to tackle the challenges associated with data multiplicity after oversampling and information loss during undersampling.
- The clustering method is employed in conjunction with oversampling and undersampling to enhance the selection of random samples for sampling and generating synthetic samples. Moreover, boosting and bagging techniques are incorporated into the learning process to enhance the overall performance of the model.
- A comprehensive set of experiments is conducted to evaluate and compare the performance of the proposed algorithm. This evaluation is carried out through comparisons with eight state-of-the-art algorithms sourced from the existing research literature. The reliability of the proposed method is assessed using datasets featuring varying imbalance ratios, and its performance is measured using multiple evaluation metrics.

Accordingly, a hybrid algorithm is proposed in this paper for imbalanced data classification where the target class of the dataset is unbalanced. Also, this algorithm tries to preserve the main features of the dataset as much as possible with modified over-sampling and under-sampling and makes predictions by applying ensemble algorithms on the balanced dataset.

## Preliminaries

In this section, a brief explanation of assumptions related to the efficacy of imbalanced data methods, and Extreme Gradient Boosting (XGBoost), bagging, and random forest algorithms is provided. These ensemble machine-learning algorithms are used in the following sections as the components of the proposed algorithm.

### Assumptions

It should be noted that the efficacy of imbalanced data methods relies on several crucial assumptions. Firstly, the assumption of minority class importance underscores the recognition that these methods prioritize the minority class, acknowledging its significance in capturing under-represented or rare events. Furthermore, the assumption regarding the representation of relevant features posits that the selected features for classification are sufficiently informative, ensuring the discernment of patterns in both minority and majority classes. The presumption of representativeness underscores that minority class instances are not mere outliers but representative of the underlying data distribution. Lastly, the assumption of independence emphasizes the expectation that instances are independent and identically distributed, acknowledging potential challenges in scenarios such as time-series or spatial data where this assumption may not always hold true. It is imperative to bear these assumptions in mind when applying imbalanced data methods, as their validity significantly influences the robustness and reliability of the results obtained.

### Extreme gradient boosting

Among various tree-based sequential models, Extreme Gradient Boosting (XGBoost) is a prominent gradient-boosting algorithm noted for its excellent accuracy and speed. In order to prevent over-fitting, XGBoost’s loss function for the objective function smooths out the final learned weights by adding an extra regularization term. The XGBoost framework is described as follows^40,41^.

The sum of the predicted score ${f}_{k}\left({x}_{i}\right)$ of all trees may be used to describe the estimated output ${\widehat{y}}_{i}$ of the gradient boosting tree model based on Eq. (1):

1 $${\widehat{y}}_{i}=\sum_{k=1}^{K}{f}_{k}\left({x}_{i}\right),\, {f}_{k} \epsilon \Gamma$$

where ${x}_{i}$ stands for the variables belonging to sample $i$ in the regression tree space, $\Gamma$ is the space of regression trees, and $K$ is the number of regression trees. Each leaf node $j$ has a prediction score ${f}_{k}\left({x}_{i}\right)$, also known as a leaf weight. The regression value for all samples at leaf node $j$ is the leaf weight ${\omega }_{j}$, where $j\in \left\{1, 2,\dots ,T\right\}$, and $T$ is the number of leaf nodes. The boosting procedure is continued until the reduction in the objective functions becomes restricted. In this algorithm, the following regularized objective function is minimized for training the model.

2 $$\Phi ={\sum }_{i=1}^{n}l\left({y}_{i},{\widehat{y}}_{i}\right)+\gamma T+\frac{1}{2}\lambda {\sum }_{j=1}^{T}{\omega }_{j}^{2}$$

In this equation, the loss function ${\sum }_{i=1}^{n}l\left({y}_{i},{\widehat{y}}_{i}\right)$ determines the difference between ${y}_{i}$ and ${\widehat{y}}_{i}$, and the regularization term $\left(\gamma T+\frac{1}{2}\lambda {\sum }_{j=1}^{T}{\omega }_{j}^{2}\right)$ penalizes the model complexity. In this regard, $n$ is the given number of observations, $\lambda$ is a regularization hyper-parameter, and $\gamma$ is the complexity cost of adding more leaves in the regularization term. In the additive learning processes, all the trees are constructed sequentially. Each recently introduced tree draws on the knowledge of its predecessors and modifies the residuals in the prediction values. Therefore, all of the trees’ iteration results have already been incorporated in ${\widehat{y}}_{i}^{\left(k-1\right)}$. As a result, the objective function at iteration $k$ is expressed as:

3 $${\Phi }_{(k)}=\sum_{i=1}^{n}l\left({y}_{i},{\widehat{y}}_{i}^{\left(k-1\right)}+{f}_{k}{(x}_{i})\right)+\gamma T+\frac{1}{2}\lambda {\sum }_{j=1}^{T}{\omega }_{j}^{2}$$

As a structural scoring function, Eq. (3) evaluates the quality of tree structure and suitability of a given vector of leaf scores where a lower number is recommended. Interested readers are referred to Chen and Guestrin^42^ for more details on the XGBoost method.

### Bagging algorithm

Bagging is one of the machine-learning techniques that combines predictors to reduce the variance and increase the accuracy of the final model. The training dataset is used to create several new datasets in the bagging algorithm, which also utilizes the bootstrapping process. This dataset is predicted by various predictors, and the final prediction is determined by voting among the predictors. In classification, the bagging approach employs voting among predictors, whereas in regression, it uses averaging among predictors^43^.

### Random forest algorithm

Random Forest is an ensemble algorithm that combines several decision trees to increase prediction accuracy. In this algorithm, sampling with replacement is used to set up separate trees with the same distribution, and then random selection is used to choose the features for each tree. Using the created trees, the final prediction is determined through voting. The random forest consists of four main steps as follows:

1. Draw random samples from the original dataset.
2. Construct an individual decision tree for each of the samples.
3. Obtain the prediction result of each of the decision trees.
4. Aggregate the results and determine the final output of the algorithm based on the majority voting for classification.

This algorithm can be used for both classification (categorical variables) and regression (continuous variables) and contains several parameters where among them, the number of decision trees in the random forest (n estimators) and the maximum number of splits that can be performed in decision trees (max-depth) are two essential parameters. The accuracy of each tree in the random forest and their interrelationships determine the error rate of a random forest. For further information on the random forest algorithm, interested readers are referred to Breiman ^44^.

## The proposed algorithm

**Figure 1:**
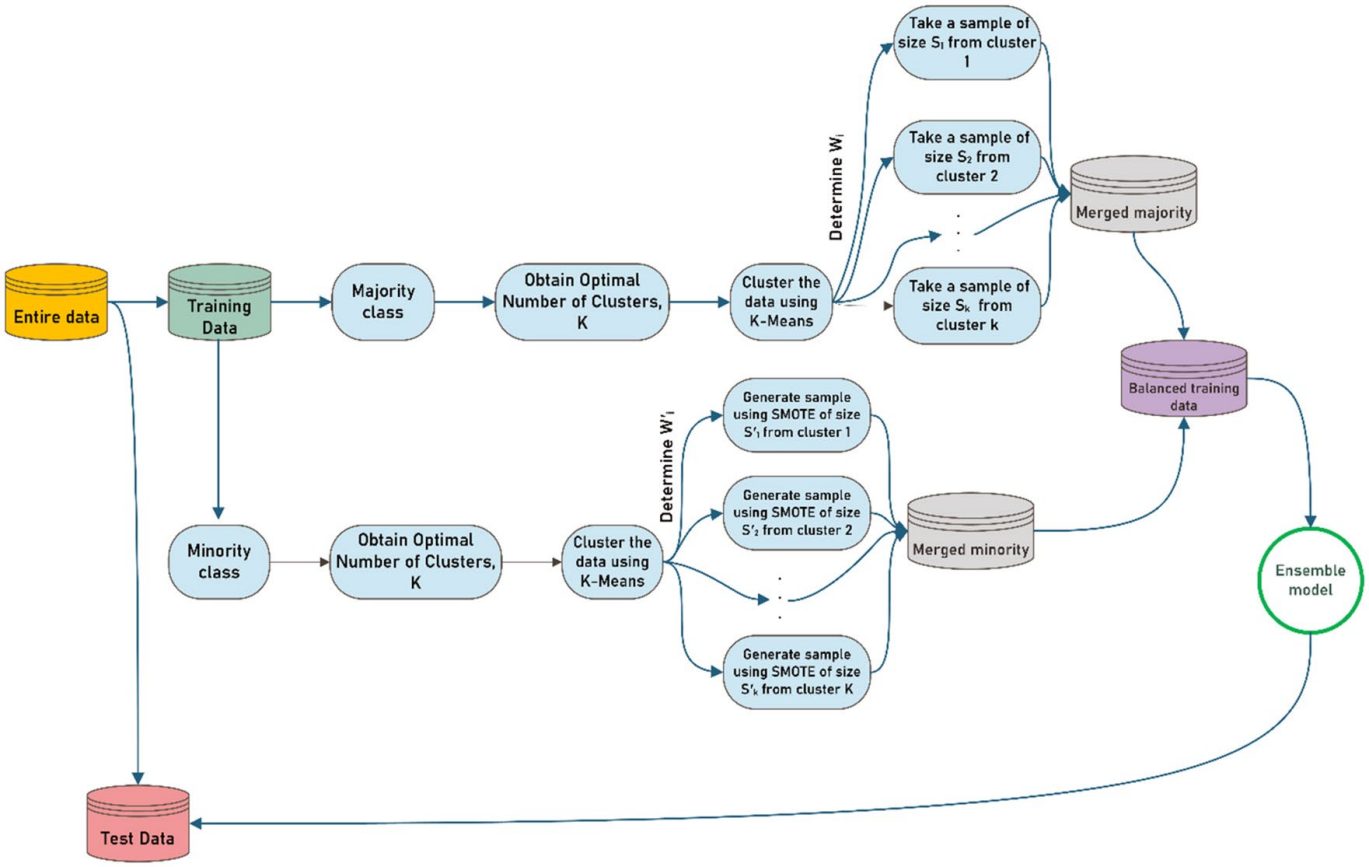
The process of the proposed CSBBoost algorithm.

In this section, a hybrid algorithm based on both unsupervised and supervised learning algorithms is proposed for the classification of imbalanced datasets. In this regard, a novel algorithm entitled cluster-based SMOTE both-sampling (CSBBoost) is proposed for classifying imbalanced data and resolving the issues with data balancing techniques. As mentioned previously, under-sampling results in the loss of much information, while over-sampling leads to a redundant increase in the size of the dataset. To address these issues, the over-sampling and under-sampling algorithms are utilized together. After balancing, the number of observations in the balanced dataset would be equal to that of the original dataset. Generating duplicated observations using minority class data leads to overfitting the training set and reducing the prediction accuracy on the test set. Hence, synthetic data is generated using the SMOTE algorithm to overcome this issue. In addition, similar observations can be grouped into some clusters based on the diversity of the data and the similarities between the observations. Considering the similarities between observations in each cluster, applying balancing techniques on each cluster would provide better results than applying them to all observations in the dataset. In addition, this clustering results in the selection of samples from the training set for both under-sampling and over-sampling that maintain the main characteristics of the dataset. The process of the proposed algorithm is represented in Fig. 1.

Based on this figure, in the first step, the data is split into two subsets, including training and test sets. Then, in the training dataset, the majority and minority classes are separated. The K-means technique is used to cluster the observations of the majority class where the appropriate $K$ for this technique is determined by the Silhouette algorithm. K-means is one of the most extensively used clustering algorithms, which divides $n$ data points into $K$ clusters to group together comparable data points, and its effectiveness varies depending on $K$. It is an iterative algorithm that allocates each data point to the cluster with the closest centroid, and the centroid of these clusters is then calculated again by taking their average. One of the most effective $K$-generating algorithms is the Silhouette coefficient which integrates both the cohesion and resolution aspects. The Silhouette for one observation $i$ is defined based on Eq. (4):

4 $$s\left(i\right)=\frac{b\left(i\right)-a\left(i\right)}{{\text{max}}\left\{a\left(i\right), b\left(i\right)\right\}}$$

where $a(i)$ is the average distance between observation $i$ and other observations in the same cluster, and $b(i)$ is the smallest average distance of observation $i$ to the observations of other clusters. If the Silhouette value is close to 1, it suggests that the observation and the cluster have a close association^45–48^. In addition, the weight parameter ${w}_{i}$ shown in Eq. (5), is generated for each cluster once the data have been clustered, according to the number of observations in each cluster.

5 $${w}_{i}=\frac{{O}_{i}}{{N}_{ma}}$$

where ${O}_{i}$ is the number of observations in cluster $i$ of the majority class, and ${N}_{ma}$ is the total number of observations of the majority class. Then, the number of observations ${s}_{i}$ for sampling from each cluster $i$ in the under-sampling algorithm is determined according to Eq. (6).

6 $${s}_{i}={w}_{i}*\frac{N}{2}$$

where ${w}_{i}$ is the weight parameter generated for cluster $i$ in the majority class, and $N$ is the total number of observations of the training set.

Similar to the dataset of the majority class, the K-means algorithm is used to cluster the training dataset of the minority class, and the Silhouette algorithm is used to find the best value of parameter $K$. Then, the weight ${w}_{i}{\prime}$ and quantity of samples ${s}_{i}{\prime}$ for over-sampling are specified according to Eqs. (7) and (8), respectively.

7 $${w}_{i}{\prime}=\frac{{O}_{i}{\prime}}{{N}_{mi}}$$

8 $${s}_{i}{\prime}=\left({w}_{i}{\prime}* \frac{N}{2}\right)-{O}_{i}{\prime}$$

where ${O}_{i}{\prime}$ is the number of observations in cluster $i$ of the minority class, ${N}_{mi}$ is the total number of observations of the minority class, and ${w}_{i}{\prime}$ is the weight parameter generated for cluster $i$ in the minority class.

The SMOTE algorithm is then used to generate new observations. SMOTE is an over-sampling technique in which, instead of duplication, the observations of the minority class are over-sampled by generating synthetic observations. Each minority class sample is over-sampled by generating synthetic samples along line segments connecting any or all of the $K$ minority class nearest neighbors. After that, observations of the minority and majority classes are merged. Finally, the random forest and Extreme Gradient Boosting (XGBoost), as ensemble learning algorithms, are used for classification due to their better performance and lower execution time. However, it should be noted that other boosting and bagging algorithms can also be applied as an ensemble model in the final step of the proposed CSBBoost algorithm. The pseudo-code of the proposed hybrid ensemble algorithm is presented in Algorithm 1.

**Figure:**
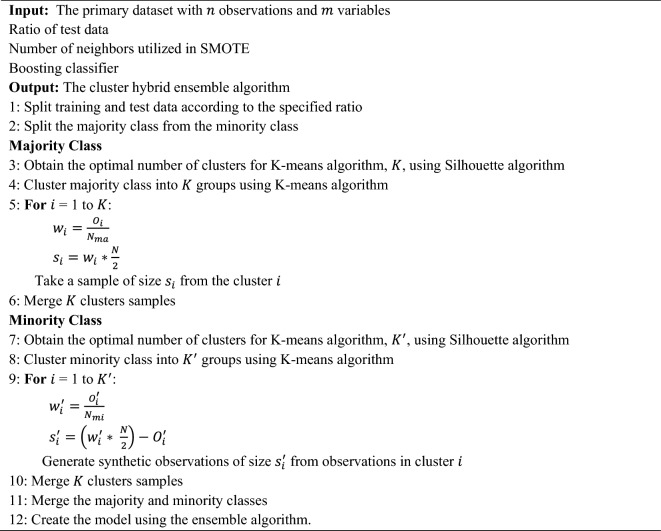
**Algorithm 1: **The proposed CSBBoost algorithm

## Performance evaluation

In this section, the performance of the proposed algorithm is evaluated and compared to several competing algorithms based on a number of datasets. In this regard, the performance measures and the results are presented in the following subsections.

### Performance measures

The most frequently used metric to determine how much a dataset is skewed is the imbalance ratio (IR) that is represented in Eq. (9):

9 $$IR = \frac{Total\, number\, of\, majority\, class\, samples}{{Total\,number\,of\,minority\,class\,samples}}$$

It should be noted that, in the case of imbalanced datasets, the cost of the misclassification of the minority class is much higher than that of the majority class in various applications, such as healthcare systems. Therefore, those performance measures would be desirable that are sensitive to both the minority and majority classes. In this regard, the F1-score is used to evaluate the performance of the proposed algorithm. F1-score is the harmonic mean of precision and recall, as follows:

10 $$F1=\frac{2 \times Precision \times Recall}{recision+Recall}$$

where the precision and recall measurements are given in the following equations:

11 $$Precision=\frac{TP}{TP+FP}$$

12 $$Recall=\frac{TP}{TP+FN}$$

Moreover, *TP* (true positive), *FN* (false negative), *FP* (false positive), and *TN* (true negative) are defined in the confusion matrix represented in Table 1.

**Table 1:** The confusion matrix.

	Predicted class	
	Positive	Negative
Actual class		
Positive	True positive (TP)	False negative (FN)
Negative	False positive (FP)	True negative (TN)

On the other hand, the Receiver Operating Characteristic (ROC) curve is a helpful tool for comparing classifiers. In this regard, the false positive rate vs. the true positive rate is plotted for various candidate threshold values between 0.0 and 1.0. The entire two-dimensional region underneath the complete ROC curve is measured by the Area Under the ROC Curve (AUC). Considering AUC as a reliable classification performance statistic, the performance of different algorithms could be compared using AUC.

### Results and discussion

In this section, the performance and effectiveness of the proposed CSBBoost algorithm are evaluated and compared to the performance of 8 hybrid data-balancing algorithms. In this regard, 20 datasets^46^ are utilized to compare the performance of the proposed algorithm to the competing algorithms, including AdaBoost^29^, RUSBoost^30^, RBBoost^31^, RHSBoost^33^, SMOTEBoost^28^, CUSBoost^32^, MEBoost^34^, and WHMBoost^35^. The details of the datasets used in the experiments are summarized in Table 2. By randomly splitting the entire dataset, 25% is used to assess the model’s performance, while 75% is used for training.

**Table 2:** Summary of dataset characteristics and imbalance ratios.

Dataset label	Dataset name	Number of variables	Number of observations	Imbalance ratio
D1	Pima	8	768	1.87
D2	Yeast1	8	1484	2.46
D3	Vehicle2	18	846	2.88
D4	Vehicle1	18	846	2.90
D5	Vehicle3	18	846	2.99
D6	Vehicle0	18	846	3.25
D7	Yeast3	8	1484	8.10
D8	Page-blocks0	10	5472	8.79
D9	Abalone9–18	8	731	16.40
D10	Yeast4	8	1484	28.10
D11	Yeast-1–2-8-9_vs_7	8	947	30.57
D12	Yeast5	8	1484	32.73
D13	Yeast6	8	1484	41.40
D14	Abalone19	8	4174	129.44
D15	Yeast-0–2-5-6_vs_3-7–8–9	8	1004	9.14
D16	Car-good	6	1728	24.04
D17	Winequality-red-4	11	1599	29.17
D18	Abalone-17_vs_7-8–9–10	8	2338	39.31
D19	Winequality-white-3_vs_7	11	900	44.00
D20	Abalone-19_vs_10-11–12–13	8	1622	49.69

The results of the proposed algorithm are illustrated and compared to the competing algorithms in Tables 3 and 4 in terms of F1 and AUC, respectively. Based on the results in Table 3, the proposed CSBBoost algorithm provides the best performance compared to other algorithms in almost all datasets. In addition, based on the results in Table 3, the proposed algorithm performs better than the competing algorithms in most cases in terms of AUC. However, in some cases, WHMBoost performs slightly better than the proposed CSBBoost.

**Table 3:** Comparative analysis of F1 scores: proposed algorithm versus competing algorithms.

Dataset label	AdaBoost	RUSBoost	RBBoost	RHSBoost	SMOTEBoost	CUSBoost	MEBoost	WHMBoost	CSBBoost
D1	0.3346	0.5543	0.3976	0.5093	0.5498	0.5543	0.2478	0.5409	**0.7712**
D2	0.5644	0.6004	0.5227	0	0.5942	0.5635	0.4264	0.5973	**0.7083**
D3	0.6425	0.7776	0.7049	0.5526	0.6605	0.7144	0.6242	0.8058	**0.9734**
D4	0.1345	0.5790	0.4917	0.1840	0.5621	0.5593	0.0371	0.5841	**0.7000**
D5	0.2145	0.5547	0.4551	0.2438	0.5368	0.5491	0.1237	0.5661	**0.6041**
D6	0.8566	0.7844	0.8139	0.6611	0.7694	0.7555	0.8555	0.8664	**0.9473**
D7	0.7029	0.6081	0.6131	0.2583	0.6217	0.6933	0.5719	0.7072	**0.8636**
D8	0.7450	0.5788	0.6274	0.5790	0.5471	0.7430	0.7314	0.7487	**0.9142**
D9	0.1883	0.2392	0.2315	0.1859	0.2395	0.2950	0.1917	0.3294	**0.5833**
D10	0.3139	0.2179	0.3074	0.0889	0.3384	0.4149	0.0590	0.3657	**0.6153**
D11	0.1620	0.1029	0.1140	0	0.1159	0.2513	0.1010	0.1326	**0.6153**
D12	0.4417	0.5320	0.6050	0.4475	0.5256	0.5879	0.2168	0.6158	**0.8695**
D13	0.0824	0.1780	0.3438	0.1693	0.3362	0.4645	0.0138	0.3618	**0.8571**
D14	**0.9959**	0.8294	0.8397	0.6533	0.8548	0.9946	0.9958	0.9898	0.4000
D15	0.4376	0.4464	0.3980	0.2014	0.4963	0.5215	0.4442	0.5310	**0.8275**
D16	0	0.2249	0.2175	0.1507	0.1631	0	0	0.3690	**0.5952**
D17	0.0495	0.1070	0.1035	0.1590	0.1212	0.0939	0.0314	0.1524	**0.6060**
D18	0.2347	0.1620	0.1686	0.0981	0.2047	0.3231	0.1672	0.2911	**0.6600**
D19	0.3069	0.0857	0.1390	0.1326	0.0953	0.2617	0.3838	0.2322	**0.6600**
D20	0.0176	0.0594	0.0821	0.0459	0.0543	0.0247	0.0124	0.1081	**0.6896**

Significant values are in [bold].

**Table 4:** Comparative analysis of AUC: proposed algorithm versus competing algorithms.

Dataset label	AdaBoost	RUSBoost	RBBoost	RHSBoost	SMOTEBoost	CUSBoost	MEBoost	WHMBoost	CSBBoost
D1	0.6334	0.6605	0.5966	0.6330	0.6640	0.6508	0.6564	0.6717	**0.8052**
D2	0.7571	0.7754	0.7582	0.4933	0.7698	0.7730	0.7694	0.7976	**0.8256**
D3	0.8302	0.9283	0.9378	0.7615	0.8760	0.9062	0.9215	0.9399	**0.9846**
D4	0.7472	0.7863	0.7663	0.4915	0.7633	0.7703	0.7960	0.8215	**0.8320**
D5	0.7085	0.7762	0.7189	0.5360	0.7534	0.7665	0.7592	0.7728	**0.7900**
D6	0.9159	0.9586	0.9706	0.8775	0.9397	0.9493	0.9713	0.9763	**0.9770**
D7	0.8744	0.9298	0.9425	0.6649	0.9200	0.9176	0.9204	**0.9557**	0.8996
D8	0.8782	0.9512	0.9562	0.9313	0.9356	0.9444	0.9489	**0.9654**	0.9330
D9	0.6095	0.7848	0.7906	0.6670	0.7692	0.7004	0.6631	0.8485	**0.8659**
D10	0.7305	0.8700	0.8636	0.6153	0.8401	0.7962	0.8042	0.8801	**0.8889**
D11	0.6028	0.6980	0.6735	0.4929	0.6460	0.6664	0.6647	0.7427	**0.7454**
D12	0.8452	0.9836	0.9692	0.9750	0.9323	0.9460	0.9593	**0.9847**	0.9517
D13	0.8518	0.9718	0.8919	0.8446	0.8794	0.8676	0.8858	**0.9210**	0.9077
D14	0.6759	0.7544	0.7469	0.6712	0.7075	0.7258	0.7227	**0.7737**	0.6200
D15	0.7511	0.7999	0.7773	0.6050	0.7794	0.7877	0.8076	0.8281	**0.8803**
D16	0.7601	0.8704	0.8514	0.7806	0.7770	0.8501	0.8735	0.9336	**0.9706**
D17	0.5873	0.6680	0.5527	0.6595	0.5981	0.5859	0.6149	0.7252	**0.7259**
D18	0.7311	0.8661	0.7984	0.7702	0.7613	0.7728	0.8093	**0.8782**	0.7548
D19	0.6039	0.8021	0.7290	0.6621	0.7281	0.6843	0.6715	0.8035	**0.9955**
D20	0.5440	0.6103	0.5985	0.5693	0.5712	0.5849	0.5878	0.6746	**0.8086**

Significant values are in [bold].

**Figure 2:**
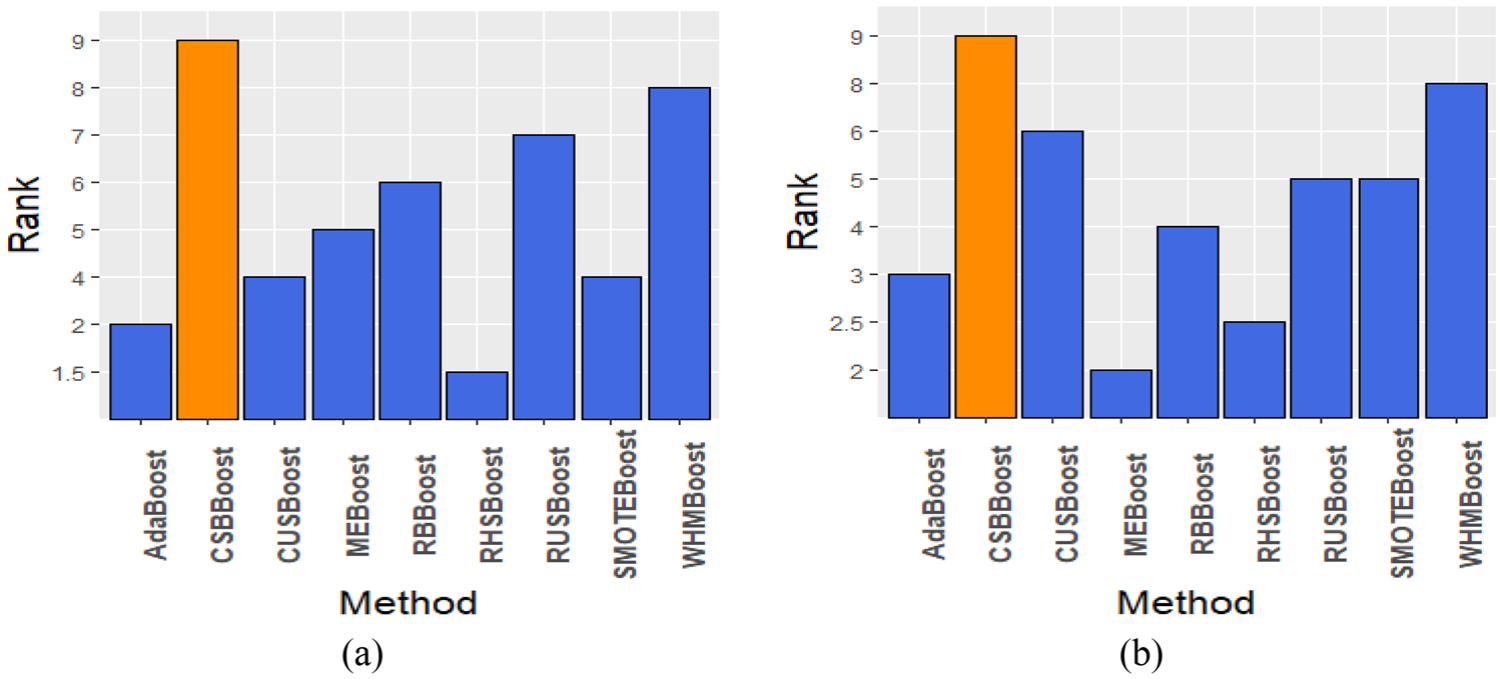
The ascending median rank of the proposed CSBBoost algorithm compared to other algorithms for (**a**) AUC, and (**b**) F1.

**Figure 3:**
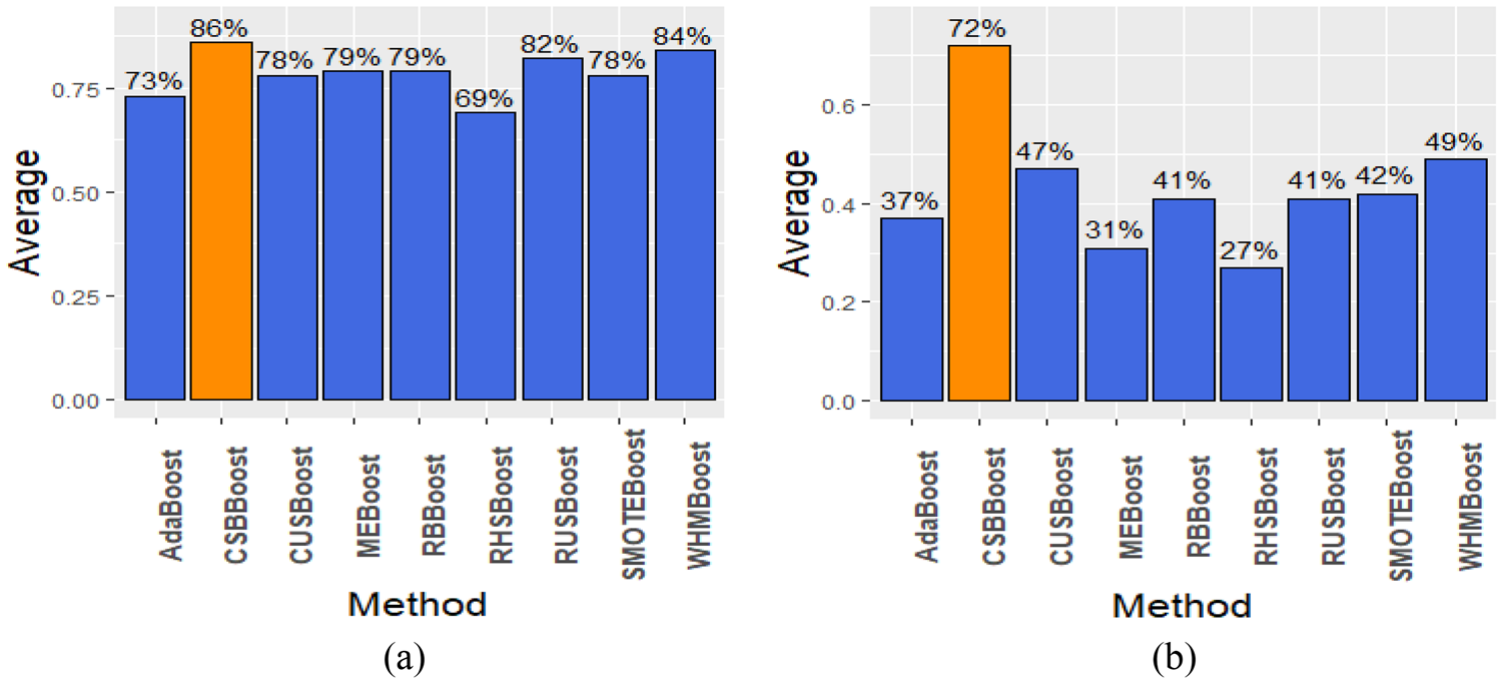
The Average of (**a**) AUC, and (**b**) F1 for proposed CSBBoost algorithm compared to other algorithms.

The performance of the algorithms is ranked based on how well they performed on each dataset, and then, the median of the ranks is used to compare their performances, considering the resistance of the median to outliers. Therefore, the proposed algorithm is compared to other algorithms based on the median of the ranks, where the ranks are sorted in ascending order, and the results are illustrated in Fig. 2. The average of F1 and AUC for various hybrid algorithms is shown in Fig. 3. This graph indicates that the proposed algorithm performs better than other algorithms.

Altogether, considering the results in Tables 3 and 4 and Figs. 2 and 3, the proposed CSBBoost algorithm is much better than the competing algorithms in terms of both F1 and AUC performance measures and under most of the datasets. However, in some cases, WHMBoost provides a slightly better AUC than the proposed CSBBoost algorithm.

## A case study

Cardiovascular diseases (CVDs) are a set of disorders that affect the heart and blood vessels, including coronary heart disease, cerebrovascular disease, congenital heart disease, rheumatic heart disease, and so on. Based on the World Health Organization (WHO) reports, 17.7 million people died due to CVDs in 2019, where 85% of these deaths were due to stroke and heart attack^49^. Some essential factors, including smoking, age, high blood pressure, and obesity, effectively identify cardiovascular patients. In addition, the Behavioral Risk Factor Surveillance System (BRFSS) is a health-related telephone survey system developed in 1984 for collecting health-related risk behaviors, chronic health conditions, and the use of preventative services. The BRFSS data integrated with other factors can be used for early diagnosis of CVDs^50^. However, the main problem in predicting CVDs is the inequality of the number of patients and healthy people where the related dataset is unbalanced, and the classifications are almost always biased towards the majority class.

In this section, the proposed CSBBoost algorithm is applied to the BRFSS dataset to demonstrate the applicability of the proposed algorithm. The BRFSS is the largest continuously conducted health survey system in the world, collecting data on American health status through yearly telephone surveys. It conducts more than 400,000 adult interviews throughout all 50 states annually. The details of the dataset and results of implementing the proposed algorithm on this dataset are provided in the following subsections.

### Dataset description

In this paper, the 2020 BRFSS dataset provided by Kaggle is used to demonstrate the applicability of the proposed algorithm. The original dataset contains 401,958 records and 279 variables^51^, where the majority of the variables inquire respondents about their health. However, the number of variables has been reduced to 18 by eliminating the less useful variables^52^. The variables in the dataset and their details are represented in Table 5. Then, a random sample of 1,000 observations is selected, and the proposed CSBBoost algorithm is applied to this dataset.

**Table 5:** Comprehensive overview of BRFSS dataset variables.

Variable	Variable type	Values/ranges	Description
HeartDisease	Categorical	No	The respondents who previously acknowledged having coronary heart disease (CHD) or a myocardial infarction (MI)
		Yes	
Body mass index (BMI)	Numerical	(12,94.8)	A measurement of body fat based on height and weight
Smoking	Categorical	No	In your lifetime, have you smoked at least 100 cigarettes?
		Yes	
AlcoholDrinking	Categorical	No	Are you heavy drinkers (men and women who consume more than 14 and 7 drinks, respectively, per week)?
		Yes	
Stroke	Categorical	No	Have you ever encountered a stroke?
		Yes	
PhysicalHealth	Numerical	(0–30)	How many days during the course of the last 30 days were you physically unwell, including any physical injuries or illnesses?
MentalHealth	Numerical	(0–30)	How many days out of the last 30 did you feel mentally unwell?
DiffWalking	Categorical	No	Do you have significant trouble climbing stairs or walking?
		Yes	
Sex	Categorical	Female	Gender type
		Male	
AgeCategory	Categorical	18–24	14 categories are used to categorize various ages
		25–29	
		30–34	
		35–39	
		40–44	
		45–49	
		50–54	
		55–59	
		60–64	
		65–69	
		70–74	
		75–79	
		80 or older	
Race	Categorical	American-Indian/Alaskan-Native	Racial/ethnicity
		Asian	
		Black	
		Hispanic	
		White	
		Other	
Diabetic	Categorical	No	Have you ever encountered a diabetes?
		No, borderline diabetes	
		Yes	
		Yes (during pregnancy)	
PhysicalActivity	Categorical	No	Adults who stated they have engaged in physical activity or exercise during the previous 30 days in addition to their usual jobs
		Yes	
GenHealth	Categorical	Poor	General state of health
		Fair	
		Good	
		Very good	
		Excellent	
SleepTime	Numerical	(1–24)	How long do you typically sleep each day?
Asthma	Categorical	No	Have you ever encountered an
		Yes	asthma?
KidneyDisease	Categorical	No	Did you ever had kidney disease, excluding kidney stones, bladder infections, or incontinence?
		Yes	
SkinCancer	Categorical	No	Have you ever encountered a skin cancer?
		Yes	

**Figure 4:**
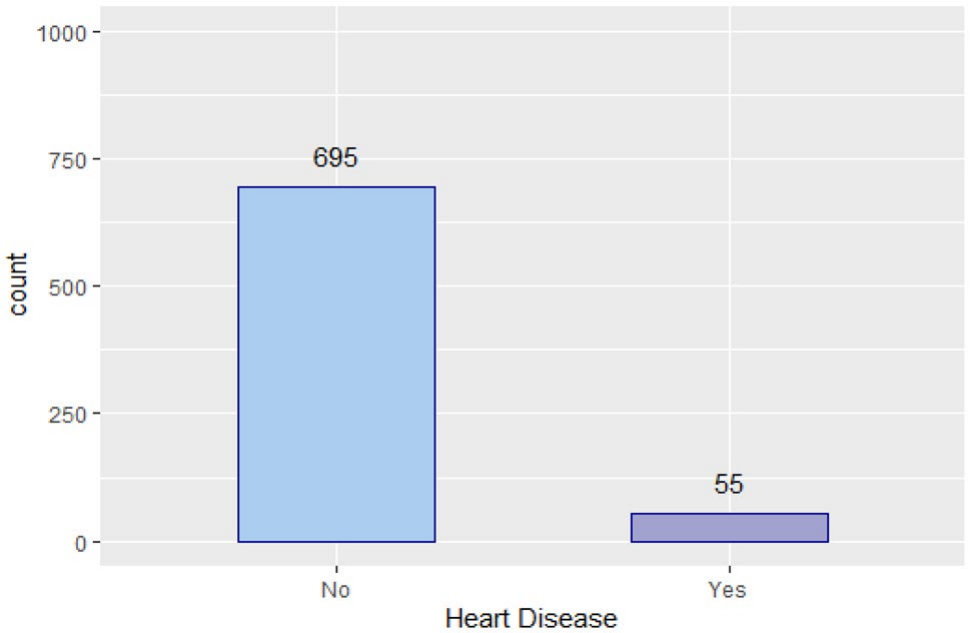
The number of patients and healthy people in training set.

The primary issue with this dataset is that the number of healthy people is higher than that of patients, which is an imbalance of the patient class. Ignoring this issue may result in inaccurate predictions. The distribution of the values of the *Heart Disease* variable in the training set is shown in Fig. 4. This figure illustrates that the *Heart Disease* variable is highly unbalanced.

### Results

**Figure 5:**
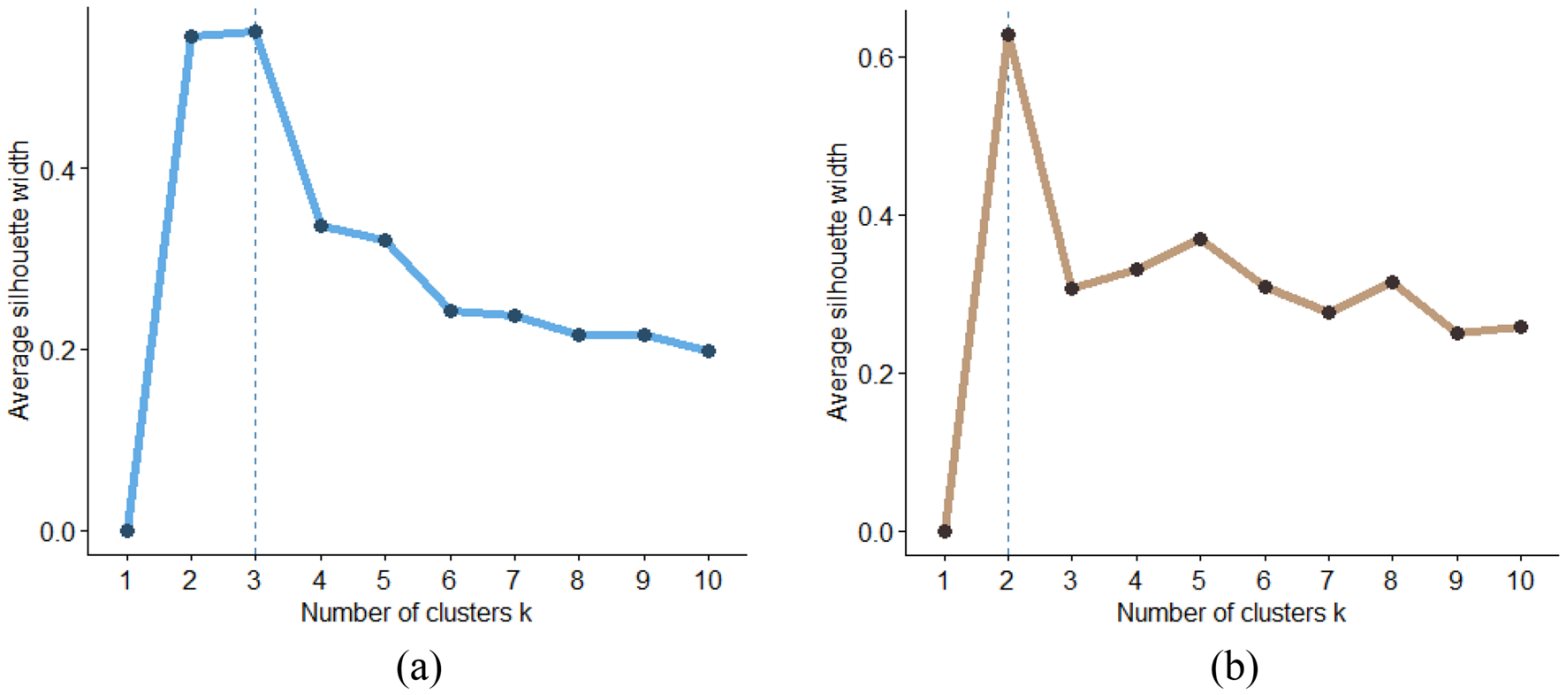
Average Silhouette width based on number of clusters for (**a**) majority class, (**b**) minority class.

The dataset is partitioned into a training set and a test set at random, where the training set is used to build the proposed model, and then, the trained model is applied to the test set. The training set comprises 75% of the total data, while the test set comprises 25%. In the following stage, the majority class is clustered using the K-Means algorithm, and the appropriate value of $K$ is determined using the Silhouettes technique. The Average Silhouette width based on the number of clusters for the majority class is shown in Fig. 5a, and the appropriate number of clusters is three.

According to Eq. (5), the weight of each cluster is determined, and Eq. (6) shows the number of samples to be taken from each cluster. Table 6 displays the weight of the clusters and the number of samples drawn from each cluster. Then, all clusters of the majority class are merged.

**Table 6:** Weight of clusters and the number of samples drawn from each cluster.

Class	Weight of each cluster (w_i_)			The number of samples taken from each cluster (s_i_)		
	w_1_	w_2_	w_3_	s_1_	s_2_	s_3_
Majority	0.128	0.791	0.080	48	296	30

The minority class is processed in the next phase. The minority class is clustered using the K-Means algorithm once the proper value of $K$ has been determined using the Silhouettes technique. Figure 5b illustrates the average Silhouette width for the minority class based on the number of clusters where two clusters are the appropriate number for clustering. According to Eq. (7), the weight of each cluster is calculated. Equation (8) estimates the number of samples that must be generated. Then, the SMOTE technique is used to generate synthetic samples where the number of nearest neighbours is considered 5. However, the value of this parameter might vary depending on the size of each dataset. Table 7 displays the weight of each cluster and the number of samples generated.

**Table 7:** Weight of clusters and the number of samples generated in each cluster.

Class	Weight of each cluster (w′_i_)		The number of samples generated in each cluster (s′_i_)	
	w′_1_	w′_2_	s′_1_	s′_2_
Minority	0.8	0.2	256	64

In the next step, all clusters of the minority class are merged. Finally, the minority and majority sets are merged to form a balanced dataset. The distribution of the values of the *Heart Patient* variable before and after balancing is shown in Table 8.

**Table 8:** The distribution of the values of the *Heart Disease* variable, before and after data balancing in training set.

Training set balance	Number (Percentage) of observations in *Heart Disease* variable	
	No	Yes
Imbalance training data	695 (92.66%)	55 (7.33%)
Balanced training data	374 (49.93%)	375 (50.06%)

The performance measures for the proposed algorithm under various ensemble prediction algorithms are shown in Table 9 (the formulas of these performance measures are given in Supplementary Appendix A). This table also includes the outcomes of using various ensemble prediction techniques on imbalanced data. The results in Table 9 illustrate that the proposed balancing technique performs better than the imbalanced dataset at detecting patients based on different performance measures. In addition, considering the F1 metric as the most important measure for identifying patients in the healthcare datasets, the proposed algorithm provides much better performance compared to the original imbalanced data.

**Table 9:** Performance comparison: CSBBoost algorithm versus original imbalanced data.

Algorithm	Accuracy	Kappa	Precision	F1	Specificity	Prevalence
CSBBoost (Gradient Boosting)	0.912	0.3073	0.30	0.3529	0.9406	0.056
CSBBoost (Random forest)	0.828	0.1850	0.40	0.2712	0.9431	0.156
CSBBoost (Bagging Tree)	0.792	0.0871	0.30	0.1875	0.9320	0.176
Original Data + Gradient Boosting	0.920	0.0775	0.05	0.0909	0.9233	0.008
Original Data + Random Forest	0.920	0.0775	0.05	0.0909	0.9233	0.008
Original Data + Bagging Tree	0.916	0.0675	0.05	0.0869	0.9230	0.012

**Figure 6:**
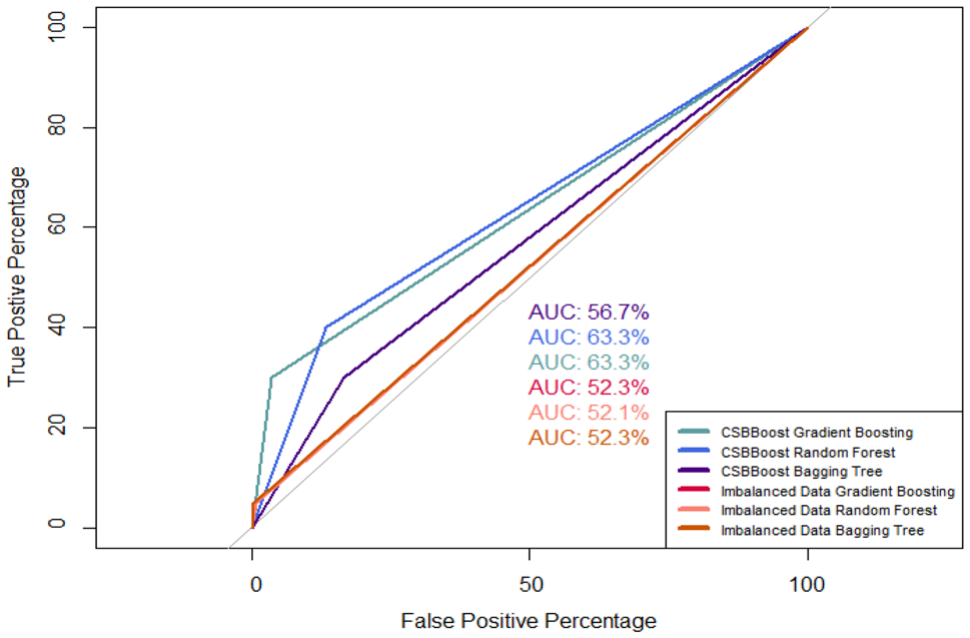
ROC curve for algorithms performed on test set.

The ROC curve of the proposed CSBBoost algorithm and Gradient Boosting, Random Forest, and Bagging tree ensemble algorithms under imbalanced data is shown in Fig. 6. According to the results, it can be concluded that the proposed CSBBoost algorithm outperforms the other algorithms and improves the performance of the algorithms under imbalanced data.

**Figure 7:**
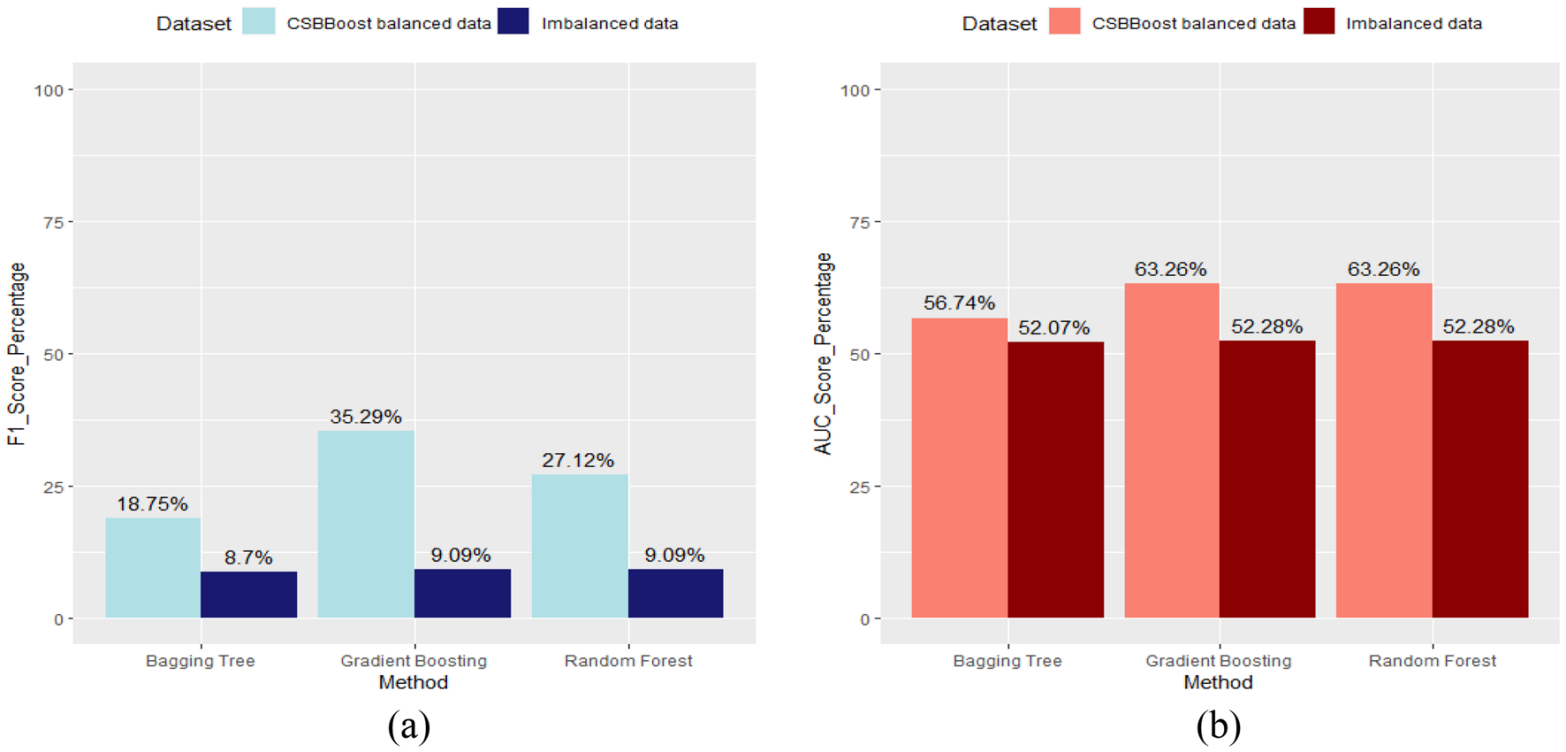
Comparing the performance of ensemble techniques on balanced and imbalanced data using (**a**) F1 and (**b**) AUC.

In addition, Fig. 7 illustrates the improvements in the prediction performance of the proposed algorithm in terms of F1 and AUC measures for each ensemble algorithm. The results in this figure demonstrate the higher accuracy and efficiency of the proposed algorithm in handling imbalanced data and identifying the patients compared to the ensemble algorithms under imbalanced data.

**Figure 8:**
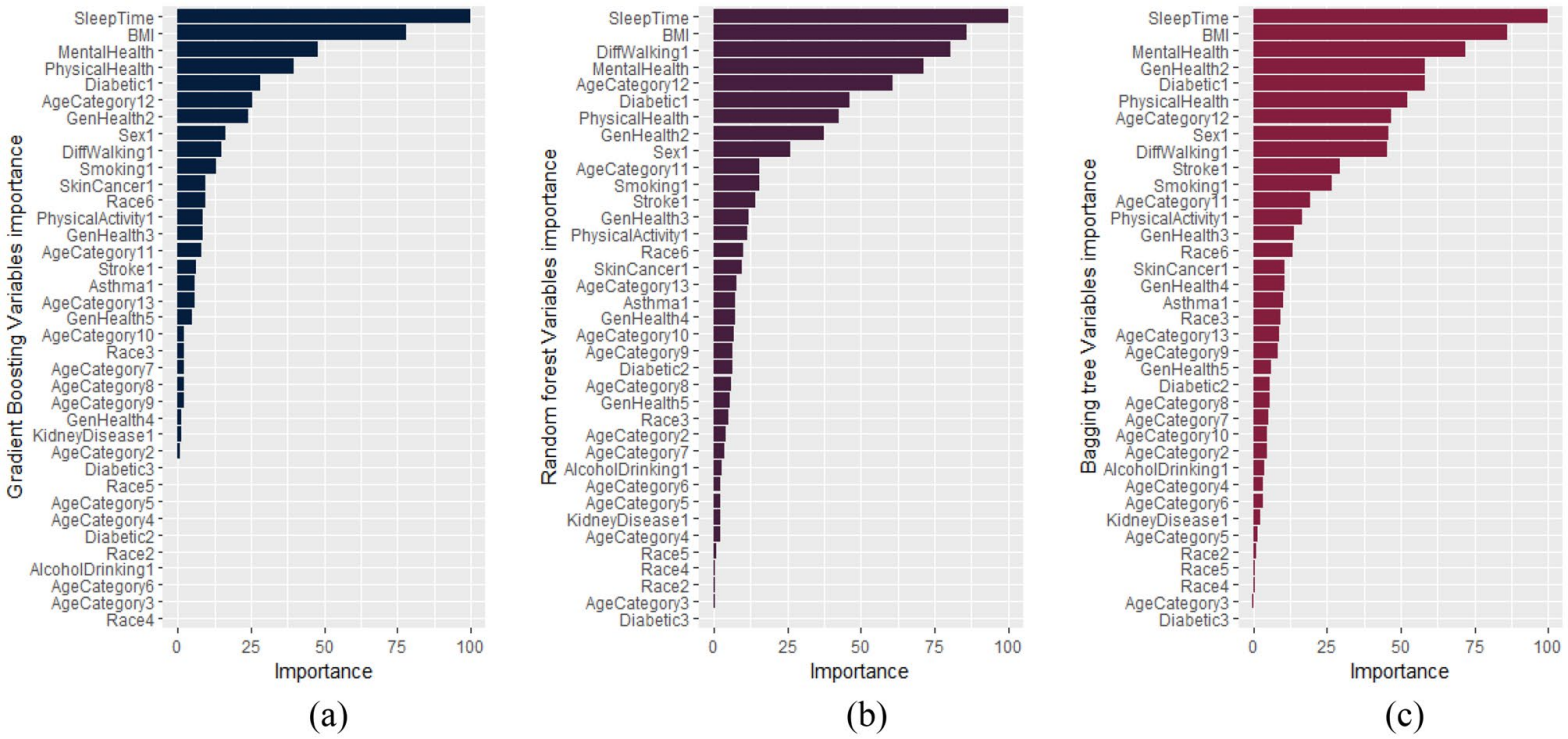
The importance of variables for boosting algorithm performed on test set for (**a**) Gradient Boosting, (**b**) Random Forest, and (**c**) Bagging tree.

Finally, Fig. 8 illustrates the importance of variables in the ensemble predicting techniques, including gradient boosting, random forest, and bagging tree. The numbers in front of some variables in Fig. 8 indicate the corresponding value/range of these variables. The order of these numbers is according to their order in Table 5.

The results in this figure demonstrate that three variables sleep time, BMI index, and mental health, are considerably effective in all three ensemble prediction models. Accordingly, these variables are significantly important in detecting cardiovascular patients.

## Conclusions

In various real-world datasets, the distribution of the class label of observations is unbalanced, and this situation leads to inaccurate predictions of the desired class and provides misleading results. Although different approaches have been proposed in the literature for data balancing, some issues exist with these approaches. Accordingly, in this paper, a cluster-based SMOTE both-sampling ensemble algorithm (CSBBoost) is proposed to eliminate the data redundancy after over-sampling, information loss after under-sampling, and improve the random selection of observations. In the proposed algorithm, the dataset is divided into the samples of the majority and minority classes, and then, the number of observations of each group is adjusted and changed to the required quantity in order to prevent redundancy. This ensures that after merging, the number of observations in the balanced dataset is equal to the ones in the original dataset. In addition, the SMOTE technique is utilized to avoid generating duplicate data by over-sampling. On the other hand, considering the randomness of samples obtained from the under-sampling and over-sampling, a clustering approach is first applied to observations, and then, the samples are obtained from each cluster to retain the characteristics of the dataset as much as possible. Finally, various ensemble algorithms, including random forest, XGBoost, and bagging, were applied for the prediction of obtained balanced dataset. The performance of the proposed CSBBoost algorithm was evaluated based on 20 imbalanced datasets and was compared with various competing algorithms, including AdaBoost, RUSBoost, RBBoost, RHSBoost, SMOTEBoost, CUSBoost, MEBoost, and WHMBoost in terms of AUC and F1. The results indicated the superiority of the proposed algorithm, where it provided much better performance than other algorithms in most cases. Finally, the performance and applicability of the proposed algorithm are illustrated through a real-world imbalanced dataset of cardiovascular heart diseases.

Some limitations of this research include the potential challenges faced by imbalanced data methods in achieving generalization across diverse domains, given variations in underlying data distributions. These methods may exhibit sensitivity to changes in data distribution over time, resulting in suboptimal adaptation and decreased performance. Additionally, the impact of noisy data, characterized by mislabeled or ambiguous instances, can significantly affect the effectiveness of imbalanced data methods, with certain techniques being more vulnerable to noise than others. Furthermore, the assumption of well-separated classes in the feature space by some methods may compromise performance in scenarios where class overlap occurs. Lastly, the performance of certain methods is highly contingent on the selection of hyperparameters, posing a complex tuning task that may demand substantial computational resources.

Imbalanced data methods in binary classification offer versatile applications across diverse domains. Their significance is notably pronounced in fraud detection, addressing the challenge of identifying rare instances of fraudulent activities amid a majority of legitimate transactions. In medical diagnosis, these techniques prove invaluable by improving the detection of rare diseases, contributing to more accurate diagnoses. Furthermore, their relevance extends to sentiment analysis, where the infrequent occurrence of specific sentiments is effectively addressed. This adaptability positions imbalanced data methods as valuable tools in enhancing precision across various critical tasks.

To enhance the proposed algorithm, it would be beneficial to delve into the integration of alternative oversampling, undersampling, and clustering methods. Improved clustering could yield superior sampling and sample generation outcomes. Furthermore, integrating cost-sensitive learning techniques into the framework, which allocates distinct misclassification costs to different classes, presents a promising avenue for future research. As datasets scale in size, the adaptability of frameworks becomes challenging; therefore, optimizing the process to achieve reduced processing times emerges as another compelling direction for future expansion.

### Supplementary Information

Supplementary Information.

## Data Availability

The datasets generated during and/or analyzed during the current study are available from the corresponding author on reasonable request.
