# Supplementary material for: A cluster-based SMOTE both-sampling (CSBBoost) ensemble algorithm for classifying imbalanced data
Source: Sci Rep. 2024 Mar 2;14:5152. doi: 10.1038/s41598-024-55598-1 (PMC10908853; doi:10.1038/s41598-024-55598-1)
Supplement: Supplementary file 1 — Supplementary Information. [file 41598_2024_55598_MOESM1_ESM.docx]

Appendix A.

The formulas of the performance measures presented in Table 9 are given as follows.

- $Accuracy=\frac{TP+TN}{TP+TN+FP+FN}$
- $Expected Accuracy=\frac{\left( TP+FN \right)*(TP+FP)}{\left( TP+FP+FN+TN \right)^{2}}+\frac{\left( FP+TN \right)*(FN+TN)}{\left( TP+FP+FN+TN \right)^{2}}$
- $Kappa=\frac{Observed Accuracy-Expected Accuracy}{1-Expected Accuracy}$
- $Specificity=\frac{TN}{TN+FP}$
- $Prevalence=\frac{TP+FP}{TP+FN+FP+TN}$
